# Screening corn hybrids for early-stage drought stress tolerance using SPAR phenotyping platform

**DOI:** 10.1270/jsbbs.23037

**Published:** 2024-07-09

**Authors:** Ajaz Ahmad Lone, Shamshir ul Hussan, Salah H. Jumaa, Zahoor Ahmad Dar, K. Raja Reddy

**Affiliations:** 1 Dryland Agriculture Research Station, Sher e Kashmir University of Agriculture Science & Technology of Kashmir, Srinagar 191132, India; 2 Department of Plant and Soil Sciences, Mississippi State University, PO Box 9555, Mississippi, 39762, USA

**Keywords:** corn, drought, SPAR, CDSRI, IDSRI, tolerant

## Abstract

An experiment was conducted comprising of six corn hybrids that were subjected to drought and irrigated environment in separate columns in soil-plant-atmosphere-research (SPAR) cubes. The treatments and hybrids in SPAR cubes were replicated four times and a two factorial randomized complete block design (RCBD) was used to analyze the effect of drought on hybrids and their effects on traits. Significant drought × hybrid interactions were observed for most of the parameters. All the traits observed under this study were affected by drought conditions. Root volume (RV) and root shoot ratio (RSR) increased, and number of root tips (NRT), number of root forks (NRF), and number of root crossings (NRC) were drastically reduced under drought conditions. The photosynthetic rate (Phot) declined by 57.96% and electron transport rate (ETR) by 54.60% and was negatively correlated with plant height (PH) and root number (RN) during drought stress. Chlorophyll content (SPAD) showed a non-significant correlation with all the traits. As per results, there were significant differences among corn hybrids for different traits studied under the SPAR setup, which indicates that this setup successfully creates differences in treatments. A cumulative drought stress response index (CDSRI) was worked out. DKC-6581 and N61X-3110 were found to be highly drought tolerant as per our findings.

## Introduction

Corn or maize (*Zea mays*), an annual C_4_ cereal that is consumed worldwide in the form of bread, whole grain, *etc*. It is used as livestock and poultry feed as well as used in biofuels. It has the highest production among all the cereals in the world and is largely a staple crop in USA. It is largely an irrigated crop in USA. However, with water availability going down through groundwater deepening and global annual temperature rise, the irrigated US corn belt in future is going to be hit hard and we might be forced to choose between drinking and irrigation water. Lessons can be learned from Africa, which is already witnessing severe droughts across all agricultural belts. Therefore, it is important to breed for drought resilience cereal for our future generations. Several drought screening methods are being employed such as response-trait-based screening and deciphering resilience mechanisms in target crops. The response traits most commonly studied are antioxidant activity, glutathione (GSH), proline response, plant growth regulator response and transcription factors based on regression studies (Hussan *et al.* 2022a). However, these studies do not elucidate an outcome where a pre-season choice can be made to decide which hybrid to grow if there is a threat of early-season drought or flowering stage drought, which is the most sensitive stage of the corn growth period. It is one of the major thrust areas of the Consortium of International Agricultural Research Centres (CGIAR) and numerous studies are being conducted worldwide. However, this experiment was laid down to screen maize hybrids at 28 days after sowing (DAS) under a simulated system called soil-plant-atmosphere-research (SPAR) (Reddy *et al.* 2001). The system was simulated to create artificial drought and irrigated conditions perfectly mimicking natural growing conditions. In this research, several physiological, root and developmental traits were analyzed under drought as well as irrigated treatments, the details of which are presented in the next section. The purpose of this experiment is to study the effect of drought on these traits of different corn hybrids at juvenile growth stages, and to perform subsequent working out of individual drought stress response index (IDSRI) and cumulative drought stress response index (CDSRI) so that these hybrids can be differentiated based on response to drought. These scientific protocols can be established to choose tolerant and resilient hybrid lines for recommendation to farmers or further experimentation for breeding.

## Materials and Methods

### Germplasm and experimental setup

**Fig. 1.**
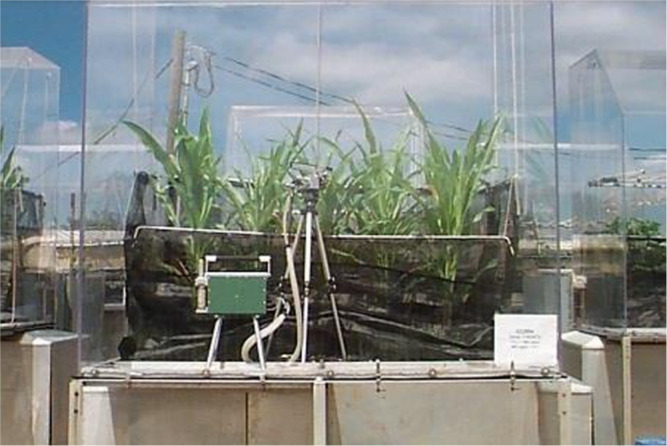
SPAR screening unit.

The experiment was conducted utilizing soil-plant-atmosphere-research (SPAR) units (Reddy *et al.* 2001) in a controlled environment facility at the environmental plant physiology laboratory, Mississippi State University, Mississippi, USA (Fig. 1). The experimental material comprised six corn hybrids *viz*. P-1498, P-1319, DKC-6581, DKC-6697, N61X-3110 and N59B-311A, which were subjected to drought as well as irrigated environment in dedicated polyvinyl chloride (PVC) columns in SPAR cubes. Each hybrid was replicated four times in both the treatments *i.e.* drought (T_1_) and irrigated/control (T_2_). Thus, making a total of forty-eight columns. The detailed layout of the experiment is given in Table 1. The experimental design followed in allocations of treatments and hybrids in SPAR cubes with two-factorial randomized complete block design (RCBD), which was intended to see the effect of drought on hybrids as well as the effect of individual hybrids on the outcome. We could work out the best hybrid performing under drought stress more precisely. The irrigated columns (T_2_) were watered three times a day using an automated drip system with full-strength Hoagland’s nutrient solution at 07:00 am, 12:00 noon and 05:00 pm. All the units were maintained at 400 ppm CO_2_ throughout the experiment. In the induced drought units (T_1_), irrigation was withheld twenty days after sowing and after that fertigation was done by real-time monitoring (Model EM50R Soil Moisture and Temperature Sensor, Decagon Devices, Inc., Pullman, WA, USA) of soil moisture status of the columns. Maintaining the moisture at 50% throughout the experiment in drought treatments (T_1_). The net solar radiation availability of 97% was maintained in the experiment units and was monitored periodically using a light meter (Li-250A, LI-COR, Inc., Lincoln, NE, USA). Temperature was maintained at 33.2°C during the day and average night temperature was maintained at 22.7°C using real-time temperature sensors.

### Measurements

#### Physiological traits

Chlorophyll content (SPAD) was worked out using a SPAD meter (SPAD 502 Minolta Inc., Ontario, Canada) at 28 DAS. Chlorophyll fluorescence (fv/fm) was calculated using Fluorpen 1000 (Photosystems instrument, Kolackova, Czech Republic). Chlorophyll fluorescence fast-transient analysis (OJIP) is an efficient non-invasive tool that measures minimal fluorescence intensity (F_o_), maximal fluorescence intensity (F_m_) and maximal variable fluorescence intensity (F_v_). It measures the fast rise of *in vivo* chlorophyll fluorescence quantum yield after incidence of photosynthetic initiating light in leaves that were dark adapted earlier. OJIP curves enable to study the changes that occur in plants at the time of light irradiance when they are suddenly flashed by fluorpen fluorometer. The F_v_/F_m_ (under dark) and F_vʹ_/F_mʹ_ (under light) or quantum yield (QY) were calculated to chalk out variable quantum efficiency of photosystem II (PSII) under both drought and irrigated conditions. Photosynthetic rate (Phot) (CO_2_ fixed per μmol m^–2^ sec^–1^) and electron transport rate (ETR) (μmol m^–2^s^–1^) were recorded by using the Li-250A (LI-COR, Inc.).

#### Developmental traits

The traits *viz*. total dry weights (TDW) (g plant^–1^), leaf area (LA) (cm^2^ plant^–1^), plant height (PH) (cm plant^–1^), leaf number (LN), shoot dry weight (SDW) (g plant^–1^), leaf dry weight (LDW) (g plant^–1^) and stem dry weight without leaves (STDW) (g plant^–1^) were measured at 28 DAS. Leaf area (LA) was measured using LI 3100 leaf area meter (LI-COR, Inc.). The dry weights were measured by oven-drying plants at 75°C until a constant weight was obtained. To account for genotypic differences, all comparisons were done with the irrigated/control treatment (T_2_).

#### Root traits

**Fig. 2.**
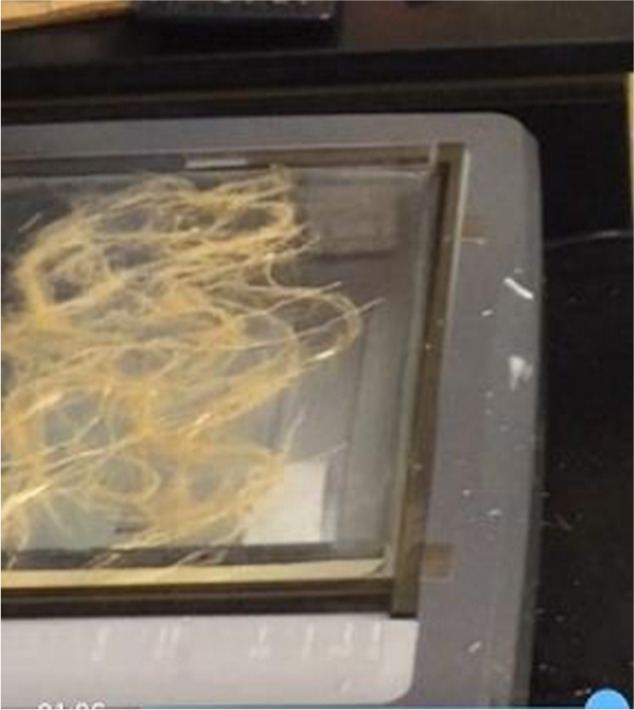
Root scanning using *WinRHIZO* optical scanner.

The following root traits *viz*. root surface area (RSA), root dry weight (RDW) (g plant^–1^), shoot dry weight (SHDW) (g plant^–1^), root/shoot ratio (RSR), total dry weights (TDW) (g plant^–1^), root length (RL) (cm plant^–1^), root diameter (RD) (mm plant^–1^), root volume (RV) (cm^3^ plant^–1^), number of root tips (NRT) (no. plant^–1^), number of root forks (NRF) (no. plant^–1^), number of root crossings (NRC) (no. plant^–1^), longest root length (LRL) (cm plant^–1^), and number of roots (RN) (no. plant^–1^) were calculated at 28 DAS. These traits were recorded after roots were separated from stems, cleaned, and disentangled using a plastic paintbrush to minimize root overlap. The cleaned roots were individually placed in 5 mm depth water in a 30 × 20 cm Plexiglas tray. The tray was placed on a dual-scan *WinRHIZO* scanner (Regent Instruments, Inc., Quebec, Canada) controlled by computer software of *WinRhizo^TM^*. Root images were obtained by setting to high accuracy resolution in gray scale image type (Singh *et al.* 2018). Images were analyzed using computer software of *XLRhizo^TM^* to obtain the above-mentioned parameters (Fig. 2).

### Data analysis, terminology and drought tolerance indices

Data collected using above-mentioned procedures was maintained in MS Excel 2016 (Supplemental Tables 1–12). Descriptive statistics including means, standard errors (SE), and ANOVA were performed using SAS software v9.4 (SAS 2011) using RCBD considering corn hybrids and treatments as sources of variation. Data was analyzed using one-way ANOVA *via* SAS GLM procedure to elucidate the effect of drought stress on physiological, developmental and root traits of corn hybrids. Fishers-protected LSD test at *p* = 0.5 was employed to test differences among treatments for different measured parameters. The standard errors of the mean were calculated using Sigma Plot 13.0 (Systat 2015) and presented in the figures as error bars.

### Drought response characterization

The six corn hybrids were divided into three drought response groups, *viz.* susceptible, moderate, and tolerant. Their individual responses were also worked out as individual drought stress response index (IDSRI) (Raman *et al.* 2012). The IDSRI were actually the ratios of trait value of the parameter in drought conditions (T_1_) to the trait value of the parameter in controlled conditions (T_2_), such that: IDSRI = P_v1_/P_o_ (where P_vl_ is trait response under drought and P_o_ is response under controlled conditions). Combined drought stress response indices (CDSRIs) were calculated by adding IDSRI for all the traits, such that: CDSRI = (PH_vl_/PH_o_) + (SPAD_vl_/SPAD_o_) + (LN_vl_/LN_o_) + (LA_vl_/LA_o_) + (LDW_vl_/LDW_o_) + (SDW_vl_/SDW_o_) + (RDW_vl_/RDW_o_) + (SHDW_vl_/SHDW_o_) + (TDW_vl_/TDW_o_) + (RS_vl_/RS_o_) + (LRL_vl_/LRL_o_) + (Fv/Fm_vl_/Fv/Fm_o_) + (TRL_vl_/TRL_o_) + (RSA_vl_/RSA_o_) + (ARD_vl_/ARD_o_) + (RV_vl_/RV_o_) + (RN_vl_/RN_o_) + (NRT_vl_/NRT_o_) + (NRF_vl_/NRF_o_) + (NRC_vl_/NRC_o_) + (Phot_vl_/Phot_o_). Based on CDSRI values corn hybrids were classified into three groups *viz*. sensitive, moderate, and tolerant.

## Results

### Performance of corn hybrids and interaction with drought

ANOVA for the traits revealed significant differences among drought/irrigated treatments for all the traits (Table 2). Significant differences were observed among corn hybrids for LA, TDW, RSA, Phot, ETR, SPAD, RSR at *p* < 0.001, RD at *p* < 0.01, RV at *p* < 0.05. The effect of corn hybrids was non-significant for the rest of the traits. The treatment × hybrid interaction was significant only for LA (*p* < 0.01) and TDW (*p* < 0.05). Pair-wise correlations at *p* < 0.001 and *p* < 0.01 were worked out for all traits under drought stress (T_1_) and controlled conditions (T_2_) (Table 4). Among physiological traits, SPAD showed a non-significant correlation with all the traits. Under drought conditions (T_1_), Phot showed a negative correlation with PH (–0.432) and RN (–0.432) at *p* < 0.01 and a positive correlation with F_vʹ_/F_m_ (0.471) at *p* < 0.01. Under control conditions (T_2_), Phot was correlated with RSR (0.829) at *p* < 0.01 and F_vʹ_/F_m_ (0.752) at *p* < 0.001. ETR was only correlated with F_vʹ_/F_m_ (0.851) at *p* < 0.001 under drought treatment (T_1_). ETR was correlated with RN (0.971) at *p* < 0.001, SHDW (0.840) at *p* < 0.01, and NRF (0.813) at *p* < 0.01 under control treatment (T_2_).

### Developmental traits

All the developmental traits *viz*. TDW, LA, PH, LN, SDW, and STDW were affected by drought, and exhibited obvious differences between control conditions (T_2_) and drought conditions (T_1_) (Table 3). Under drought conditions (T_1_), PH ranged from 24.50 cm (P-1498) to 19.25 cm (DKC-6697) with an overall average of 22.04 cm. The average PH under the control treatment was 37.29 cm. A maximum PH reduction of 17.5 cm was observed in DKC-6697. LA was also drastically reduced under drought conditions (T_1_). It ranged from 2600.07 cm^2^ (P-1498) to 1787.60 cm^2^ (N59B-311A) under control conditions and ranged from 563.28 cm^2^ (P-1319) to 601.94 cm^2^ (N59B-311A) under drought conditions (T_1_). Maximum LA reduction was observed in P-1498 (76.88%) whereas minimum LA reduction of 66.32% was observed in N59B-311A. LA varied under drought treatment (T_1_) neatly corresponding to LDW. LDW also observed a similar trend to LA, that maximum LDW reduction was observed in P-1498 and minimum LDW reduction was observed in N59B-311A. Thus, there was a coherence of TDW and LDW in the same genotype. Under drought conditions (T_1_), LN ranged from, 5.25 (DKC-6581 and P-1498) to 4.75 (DKC-6697 and N59B-311A). The average LN under the control treatment (T_2_) was 6.458. LN reduction was 1.5 in all hybrids except P-1498 in which it was only 1.25. Under drought conditions (T_1_), RSR changed significantly from control with an average increment of 233%. The minimum RSR under drought was expressed by N59B-311A (0.32) and the maximum P-1498 (0.38), with an average value of 0.353 as compared to 0.106 under control conditions. Both SDW and SHDW were affected by drought stress, however, the reduction due to drought was more drastic for SHDW. SDW exhibited an average decline of 65.18% with maximum reduction in DKC-6581 and minimum reduction in N59B-311A hybrid. Whereas average decline of SHDW under drought stress was 67.20% with a maximum reduction in DKC-6581 and minimum reduction in N59B-311A. SDW and SHDW also exhibited coherence in the same genotype.

### Root traits

All the root traits were affected by drought, but some hybrids managed to reduce the disparity between drought conditions (T_1_) and control conditions (T_2_) treatments for some root traits (Table 3). LRL ranged from 61 cm in N59B-311A to 66.75 cm in P-1498 under drought treatment (T_1_), and from 55.75 cm in N59B-311A to 59.25 cm in P-1498 under control treatment (T_2_). The average increase in LRL due to drought stress was 10.50%. Under control treatment (T_2_), RN ranged from 17.25 in DKC-6581 to 14.00 in P-1319 with an overall average of 15.66. Under drought treatment (T_1_), RN ranged from, 10 (DKC-6697) to 11.75 (P-1498). The average RN under drought treatment (T_1_) was 10.95. Maximum RN reduction due to drought was observed in DKC-6581 and minimum was observed in N59B-311A. The average decrease in RN of all hybrids due to drought stress was 30%. RDW increased due to drought stress and ranged from 1.13 in N59B-311A to 1.38 cm in P-1498 under drought treatment (T_1_) and from 0.94 in N59B-311A to 1.29 in DKC-6581 under control treatment (T_2_). A minimum difference in RDW under control (T_2_) and drought treatment (T_1_) was observed in DKC-6697. RSA was also affected by drought, and it tends to decrease when under drought. However, in two hybrids DKC-6581 and DKC-6697, the decrease was rather drastic 33.47% and 35.19%, respectively. RD generally increased due to drought stress. However, it unchanged in DKC-6697 (0.52 mm) and N59B-311A (0.49 mm). TRL was reduced under drought stress in all hybrids. It ranged from 4507.91 cm in P-1498 to 5220.65 cm in P-1319 under drought conditions (T_1_) with an average TRL of 4895.81 cm. The average TRL under control treatment (T_2_) was 6798.74 cm. The percentage decrease in the average TRL due to drought stress across all hybrids was 27.98%. Minimum decline in TRL was observed in N61X-3110 (22.26%). RV ranged from 7.78 cm^3^ plant^–1^ in P-1498 to 10.12 cm^3^ plant^–1^ in N61X-3110 under drought conditions (T_1_) with an average RV of 9.12. The average RV under control treatment (T_2_) was 11.05 cm^3^ plant^–1^. Overall average decline under drought conditions (T_1_) was observed but the RV increased under drought (T_1_) in P-1498 from 6.66 to 7.78 cm^3^ plant^–1^. NRT, NRF and NRC were drastically reduced under drought conditions (T_1_). NRT ranged from 32207.25 in DKC-6697 to 42571.75 in DKC-6581 under control conditions (T_2_) and ranged from 21485.75 in P-1498 to 28464.25 in DKC-6581 under drought conditions (T_1_). Maximum NRT reduction was observed in P-1498 (44.76%) whereas minimum NRT reduction of 27.80% was observed in DKC-6697. NRF ranged from 49278.50 in P-1498 to 79550.25 in DKC-6581 under control conditions (T_2_) and ranged from 37754.75 in P-1498 to 48596.25 in N61X-3110 under drought conditions (T_1_). Maximum NRF reduction was observed in DKC-6697 (47.01%) and DKC-6581 (46.7%) whereas minimum NRF reduction of 23.38% was observed in P-1498. NRC ranged from 4905.00 in P-1498 to 6115.75 in DKC-6581 under control conditions (T_2_) and ranged from 2728.00 in DKC-6697 to 3469.00 in N59B-311A under drought conditions (T_1_). Maximum NRC reduction was observed in DKC-6581 (54.15%), whereas minimum NRC reduction of 32.34% was observed in N59B-311A.

### Physiological traits

Mean values of traits *viz*. SPAD, F_v_/F_m_, Phot and ETR for all hybrids under drought treatment (T_1_) and control treatment (T_2_) are presented in Table 3. SPAD values of all the hybrids declined due to drought treatment. Under drought conditions (T_1_), minimum SPAD value was observed at 30.15 in DKC-6697 and maximum SPAD value was recorded at 35.93 in N61X-3110 and the overall average SPAD value for six hybrids was 32.85. Under control treatment (T_2_), it ranged from 36.30 in DKC-6697 to 43.33 in DKC-6581 with the overall average standing at 39.86. The average SPAD decline was 17.5% due to drought stress. F_v_/F_m_ was also reduced due to drought stress. The average F_v_/F_m_ under control treatment was 0.455 and 0.32 under drought. The mean reduction of F_v_/F_m_ due to drought was 29.67%. Phot was reduced by more than 50% under drought treatment in all hybrids. It ranged from 15.03 in N59B-311A to 18.20 in DKC-6581 under drought stress with an overall average of 16.05. The average Phot under the control treatment was 38.18. The average Phot decline from control treatment (T_2_) to drought treatment (T_1_) was 57.96%. A similar trend was observed in ETR. The range of ETR across hybrids under drought treatment (T_1_) was 77.42 in P-1319 to 109.17 in DKC-6697 with an average of 96.13, whereas the average ETR under control treatment (T_2_) was 211.73. The average decline in ETR from control to drought was 54.60%.

### Root traits using image analysis

**Fig. 3.**
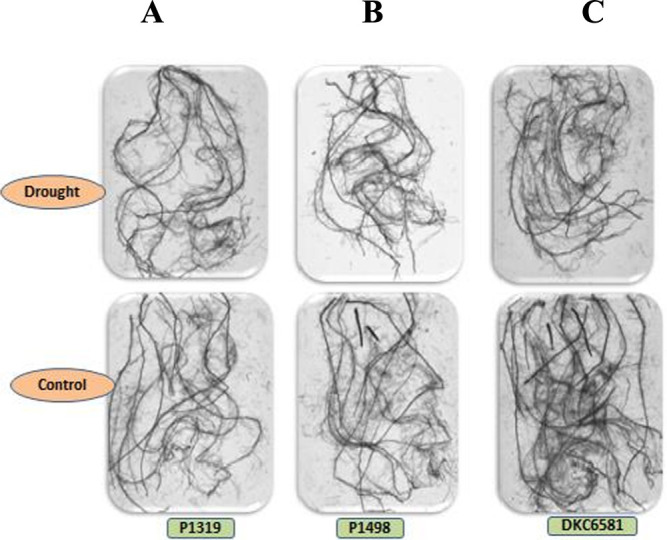
Representative scanned root images from controlled and drought sets in each drought stress response classes: (A) sensitive reaction (B) moderate reaction, and (C) tolerant reaction groups.

A scanner-based root imaging technique was used to visualize and understand the root structure of six maize hybrids under drought (T_1_) as well as irrigated/control (T_2_) treatments. Upon examination of carefully harvested roots under the *WinRhizo* scanner setup, it was observed that there is a corresponding concurrence between the early vigor parameters of the hybrids and scanned images of the roots. Therefore, it is an indication that CDSRI values are not hearsay and may interestingly reflect the overall response of each hybrid under drought stress (Fig. 3). DKC-6581 and N61X-3110 exhibited robust root structure with good RN and LRL, whereas P-1319 and N59B-311A had a weak and unimproved root structure under drought treatments (T_1_) (Fig. 3).

### Classification of corn hybrids based on drought stress indices

By combining physiological, developmental and root traits using a simulated SPAR apparatus, an early drought stress scoring system for corn might be developed through this study. CDSRI and IDSRI values were used to classify these six corn hybrids into sensitive, moderate, and tolerant groups of drought stress responses. CDSRI values ranged from 25.21 (highly sensitive) in P-1319 to 28.32 (highly tolerant) in DKC-6581 (Table 5). Pairwise correlations among the traits were calculated to assess the extent of interrelationships between the traits as continuous variables, which could potentially serve as a basis for the indirect selection of promising hybrids.

## Discussion

Corn is grown worldwide in irrigated, rainfed and dryland areas, however most of the cultivated varieties are hybrids. Therefore, characterization of corn hybrids for drought stress tolerance-related traits is of utmost necessity. This study is the first to screen corn hybrids grown in a modern phenotyping platform for drought-related traits. The variable response of corn hybrids to drought-induced stress can be assessed through this SPAR apparatus for commercial purposes as well as to unravel *per se* resilience response. Seed providers can screen hybrids suited for drought affected areas, limited irrigation locations and dry farming areas not only in the USA but throughout the maize farming areas in the world. Various drought related studies have been performed in corn lines, varieties and hybrids but a sophisticated drought simulation study concluding by 28 DAS is novel and very helpful approach in screening a large population within a limited time. Also, the physiological outcome of corn hybrids in such a setup opens the doors for understanding the water requirement at specific stages, which leads to precise control of water regimes. SPAR setup can be used close to a commercial or a domestic corn field to observe the water requirement of the experiment; so that water can be applied or provided to the main field as per the inferences drawn from the SPAR block.

### Performance of corn hybrids and interaction with drought

Drought stress variably influences different stages of the crop life cycle thus producing variable drought stress responses under diverse environments. Identifying the combinations of favorable shoot morphological, physiological, and root traits can lead to sustainable corn yields under drought stress. The analysis of variance depicted that all the traits were significantly affected by drought stress. However, there was significant effect of hybrids only on LA, TDW, SPAD, Phot, ETR, RV, RD, and RSR. Hybrid-treatment interaction was found significant only in TDW and LA. As per the ANOVA, significant variations for all the traits due to the drought effect and hybrid effect were observed. It indicated the varied response of hybrids due to drought stress. Additionally, variation in the inherent genetic setup of the hybrids can also be inferenced from these findings. This kind of experiment can be laid down to evaluate corn inbreds for selection in hybrid development and other breeding programs. Traits such as PH, rooting depth, RD, and LA, have been known to be influencing drought tolerance in corn (Kamoshita *et al.* 2008). Therefore, correlation analysis becomes important to understand the true relationship between vegetative traits and yield components under drought. This way we can understand the extent to which these traits are affecting drought tolerance. Therefore, understanding the relationship between traits can also throw a light on the direction of selection and narrows down the number of traits that can be looked upon as significant and important for further elucidation. However, after it is determined that a given number of traits determine yield output under drought stress, care should be given to the fact that these should also be phenomenally convenient to score or measure (Wijewardana *et al.* 2015).

### Developmental traits

Drought stress can have serious implications on plant growth parameters, however, some genotypes/varieties succeed in mitigation and hence tend to optimize their growth in various trait departments (Hussan *et al.* 2022b) on the hind sight they may fail in some other traits as well. In this study, it was observed that as per ANOVA, all the developmental traits were affected by drought. These findings are in agreement with Bota *et al.* (2004), who indicated that under waterless conditions, cell division and cell elongation are affected during the early vegetative phase of crop growth. In the present research experiment, LA reduction due to drought stress was significantly visible and might be attributed to reduced *RuBisCO* activity during water deficit (Loreto *et al.* 1995). Under drought stress, changes in P3 and the quantity of CO_2_ in the chloroplast are detrimental to the overall photosynthetic activity of the plant (Borrell *et al.* 2000). It is well known that leaf senescence happens due to drought stress. The ability of a plant to maintain green cover, which is measured as leaf area index is an interesting benchmark to understand the performance of a variety under drought stress (Farooq *et al.* 2010, Murty and Murty 1982). A reduced leaf area index affects the total dry matter accumulation in plants due to reduced photosynthetic area and subsequently photosynthetic activity. Therefore, under drought stress negatively affected dry matter accumulation as a consequence of diminished leaf area index which indicates the effect of genotype (Mostajeran and Rahimi-Eichi 2009, Xu *et al.* 2015). In the present investigation, the average increase in root-shot ratio from irrigated treatment (T_2_) to drought treatment (T_1_) was 233%. It has been reported that drought stress induces rapid transport of sucrose from the leaves to the roots, which is due to the overproduction of sucrose phosphate synthase in the leaf tissues and root invertase enzyme in the roots. Elevated activity of these enzymes and subsequent sucrose transport to roots enhances dry matter production in roots (Yoshida and Hasegawa 1982). However, the real purpose of this study is to decipher the degree of effect that drought might have on the trait parameters of corn hybrids. It should be expected that genotypic differences would kick in and there is always scope for differential response by different hybrids to drought stress. Maximum LA reduction due to drought was observed in P-1498 (76.88%), whereas minimum LA reduction of 66.32% was observed in N59B-311A. LA varied under drought treatment (T_1_) neatly corresponding to LDW. LDW also exhibited a similar trend to LA, that maximum LDW reduction was observed in P-1498 and minimum LDW reduction was observed in N59B-311A. Thus, there was a coherence of TDW and LDW in the same genotype indicating the relatedness of these traits. SDW exhibited maximum reduction due to drought in DKC-6581 and minimum reduction in N59B-311A hybrid. A similar trend was shown by SHDW. Therefore, these two traits also showed coherence in the same genotype.

### Root traits

Roots are the primary appendage of a plant to fetch nutrients (Paez-Garcia *et al.* 2015). Variances in root traits such as RL, RD and root branching are directly detrimental to the magnitude of nutrient and water uptake, photosynthetic activity and consequently, biomass produced by the plant (Kano-Nakata *et al.* 2011, Tran *et al.* 2015). All the root traits were affected by drought, but some hybrids managed to reduce the disparity between drought (T_1_) and control treatments (T_2_) for some root traits. The average increase in LRL due to drought stress was 10.50%. It has been revealed that after a root attains a certain length and thickness the process of lateral root development starts from the root pericycle (Abe and Morita 1994). And, it is the lateral roots that perform a majority of nutrient and water absorption (Yoshida and Hasegawa 1982), because 70 percent of total RSA comes from the lateral roots (Parker *et al.* 2000). In this study, the average decrease in RN of all hybrids due to drought stress was 30%. RDW was also reduced due to drought however, the values under drought (T_1_) and irrigated conditions (T_2_) remained visibly comparable. It is remarkable to note that the RD is a function of cell elongation and cell elongation is known to increase under drought stress. The reduction in cell division coupled with enhanced cell elongation kept drought root diameter phenotype comparable to that of irrigated roots (Samson and Wade 1998). Apart from RL and RD, the optimization of root architecture is also important for efficient water and nutrient absorption. It is also important to develop forks, tips, and crossings for penetrating, grabbing, and covering the soil surface area efficiently. Therefore, the study of such parameters helps us to understand the behavior and performance of hybrid varieties for consideration in cultivation. In the present investigation, significant variation for these three traits was observed in our test hybrids. In water-stressed soils, there is reduced oxygen supply, with a physical barrier such as hardpans, and generally poor adaptation of roots to the aerobic condition. These in turn limit the exploitation of deeper soil layers, hence reducing root length and biomass production (Clark *et al.* 2008). NRF was also correlated with ETR, which indicates the importance of forks, tips, and crossings.

### Physiological traits

Whether it is the physical growth or yield reduction, the path of such responses is always physiological, which ultimately results in chlorophyll reduction and under performance of photosynthetic machinery (Fu and Huang 2001). The multiplicity of crop growth factors involved in drought stress injury suggests that screening studies might be useful for characterizing drought resistance (Hura *et al.* 2007). As per Table 3, significant differences of SPAD values between well-watered (T_2_) and water-stressed (T_1_) conditions were observed. The average SPAD decline due to drought was 17.5%. SPAD reading is a measure of chlorophyll content, which is an outcome of drought stress-induced photo bleaching and photo-inhibition (Long *et al.* 1994), and it is a lucrative scale for screening of hybrids for water and drought stress tolerance (O’Neill *et al.* 2006). F_v_/F_m_ is the measure of chlorophyll fluorescence and a decline in this value indicates photo-oxidative stress damage to the PSII (Ambavaram *et al.* 2014, Maxwell and Johnson 2000). In this study, the mean reduction in F_v_/F_m_ due to drought was 29.67%. The ability to maintain high F_v_/F_m_ under drought stress thus indicates a high efficiency of radiation use, possibly for photochemistry and carbon assimilation. In weeping *lovegrass* high F_v_/F_m_ value is known to be positively correlated with drought tolerance and low F_v_/F_m_ is a feature of drought susceptible genotypes when subjected to drought (Colom and Vazzana 2003). Individually, F_v_ and F_m_ values under both control (T_2_) and drought conditions (T_1_) were statistically significant for cultivars as well as for their genotype-drought interactions.

### Identifying drought tolerant corn hybrids

Contemporary methods of evaluation of genotypes for abiotic stresses including drought are not specially developed for stress-based evaluation (IRRI 2014). They are simply a part of basic genotype evaluation studies. Therefore, a method or scale is required that can give a broader and precise picture of the performance of a genotype/variety during drought stress. The CDSRI values calculated under this study using early season screening in SPAR tubes gave a very clear picture of the performance of the hybrids, because these values combine the effect of all the traits to give a conclusive picture of the performance of test lines/genotypes/hybrids. In the present study, DKC-6581 and N61X-3110 were found to be highly drought tolerant as per our findings. Thus, they might be used along with other water-saving strategies to improve crop yields in commercial crop production. As per our findings, there were significant differences among corn hybrids for different traits studied under the SPAR setup, which indicates that this setup successfully creates differences due to the treatments. It is further recommended that the SPAR setup should be used for the evaluation of maize inbred lines rather than the hybrid, which would help in the usage of inbred lines in hybrid development.

## Author Contribution Statement

A.A.L., field experiment and data collection, S.U.H., framing, writing, data interpretation, revisions and final editing, S.H.J., laboratory studies and data analysis, Z.A.D., interpretation and drafting, K.R.R., experimental material, resources, supervision of experiment.

## Supplementary Material

Supplemental Tables

## Figures and Tables

**Table 1.** The layout of the experiment in SPAR

SPAR Pot NO.	45	41	37	33	29	25	21	17	13	9	5	1
Variety	N59B-311A	DKC-6697	P-1319	DKC-6697	P-1319	DKC-6581	N59B-311A	P-1498	DKC-6581	N59B-311A	DKC-6697	DKC-6581
SPAR Pot NO.	46	42	38	34	30	26	22	18	14	10	6	2
Variety	N61X-3110	P-1498	DKC-6581	P-1498	N61X-3110	N59B-311A	P-1498	N59B-311A	N61X-3110	P-1319	P-1319	P-1498
SPAR Pot NO.	47	43	39	35	31	27	23	19	15	11	7	3
Variety	P-1498	DKC-6581	N61X-3110	P-1319	N59B-311A	P-1498	N61X-3110	DKC-6697	P-1319	DKC-6697	P-1498	N59B-311A
SPAR Pot NO.	48	44	40	36	32	28	24	20	16	12	8	4
Variety	P-1319	DKC-6697	N59B-311A	DKC-6581	N61X-3110	DKC-6697	DKC-6581	P-1319	DKC-6697	DKC-6581	N59B-311A	N61X-3110

**Table 2.** ANOVA across the genotype, treatments, and their interaction for the morphological parameters measured *viz.* PH (cm plant^–1^), LA (cm^2^ plant^–1^), RSA (cm^2^ plant^–1^), RSR, TDW (g plant^–1^), RL (cm plant^–1^), RD (mm plant^–1^), RV (cm^3^ plant^–1^), NRT (plant^–1^), NRF (plant^–1^), NRC (plant^–1^), SPAD, Phot, ETR and F_v_/F_m_. Measurements were measured at the harvesting time, 28 DAS

Source	PH	LA	TDW	RL	RSA	RD	RV	NRT	NRF	NRC	Photo	F_vʹ_/F_mʹ_	ETR	SPAD	RSR
Treatment	***	***	***	***	***	**	*	***	***	***	***	***	***	***	***
Hybrid	NS	**	*	NS	**	*	*	NS	NS	NS	***	NS	*	***	NS
Treatment × Hybrid	NS	**	*	NS	NS	NS	NS	NS	NS	NS	NS	NS	NS	NS	NS

***, **, *, and NS mean significance levels at *p*-value < 0.001, 0.01, 0.05, and not significant, respectively.

**Table 3.** Drought stress effects on morpho-physiological parameters measured at the harvesting time, 28 DAS. PH (cm plant^–1^), LN (no. plant^–1^), LA (cm^2^ plant^–1^), LDW (g plant^–1^), SDW (g plant^–1^), RSA (mm^2^ plant^–1^), RDW (g plant^–1^), SHDW (g plant^–1^), RSR, TDW (g plant^–1^), RL (cm plant^–1^), RD (mm plant^–1^), R (cm^3^ plant^–1^), NRT (no. plant^–1^), NRF (no. plant^–1^), NRC (no. plant^–1^), LRL (cm plant^–1^), RN (no. plant^–1^), SPAD, Phot, ETR and F_v_/F_m_

Trait	P-1498			P-1319			DKC-6581			DKC-6697			N61X-3110			N59B-311A	
	C	D		C	D		C	D		C	D		C	D		C	D
PH	38.75	24.50		36.25	22.25		37.50	22.00		36.75	19.25		38.75	23.00		35.75	21.25
LA	2600.07	600.93		2033.23	563.28		2353.26	582.54		2339.89	564.03		2128.37	569.95		1787.60	601.94
LN	6.50	5.25		6.50	5.00		6.75	5.25		6.25	4.75		6.50	5.00		6.25	4.75
SPAD	39.60	32.85		38.55	30.48		43.33	34.60		36.30	30.15		41.83	35.93		39.58	33.10
LRL	59.25	66.75		56.00	65.00		58.25	62.25		55.75	61.50		57.50	62.00		55.75	61.00
RN	14.25	11.75		14.00	11.00		17.25	10.75		16.75	10.00		16.00	10.75		15.75	11.50
LDW	7.63	2.17		6.66	2.06		7.91	2.29		7.22	2.09		6.42	2.28		5.20	2.17
SDW	3.75	1.63		3.57	1.39		5.00	1.43		4.62	1.33		4.18	1.40		3.20	1.33
SHDW	11.39	3.80		10.63	3.45		12.91	3.73		11.83	3.42		10.59	3.65		8.40	3.50
RDW	1.15	1.38		1.09	1.29		1.29	1.21		1.22	1.15		1.22	1.14		0.94	1.13
RSR	0.10	0.38		0.10	0.37		0.11	0.36		0.11	0.34		0.11	0.35		0.11	0.32
TDW	12.10	4.88		11.71	4.74		14.20	4.93		13.24	4.57		11.81	4.49		9.33	4.63
RSA	705.54	663.65		945.96	811.77		1116.13	742.54		1105.68	716.50		983.60	810.23		916.94	740.31
RD	0.37	0.47		0.43	0.49		0.46	0.49		0.52	0.49		0.46	0.50		0.47	0.47
RL	6025.57	4507.91		7041.50	5220.65		7748.80	4791.84		6752.40	4613.85		6693.33	5202.80		6531.25	5037.82
RV	6.66	7.78		10.15	10.06		12.99	9.16		14.46	8.95		11.61	10.12		10.48	8.67
NRT	38899.25	21485.75		40179.75	23325.75		42571.75	28464.25		32207.25	23252.50		33353.00	23283.75		41103.75	25777.25
NRF	49278.50	37754.75		64806.00	47135.50		79550.25	42397.25		73482.25	38932.25		69277.75	48596.25		65371.50	47962.25
NRC	4905.00	2871.00		5707.75	3363.25		6115.75	2803.75		5467.00	2728.00		5630.75	3143.00		5127.25	3469.00
Phot	35.83	16.20		30.48	15.75		42.13	18.20		36.98	15.25		42.78	15.88		40.90	15.03
F_v_/F_m_	0.49	0.30		0.45	0.29		0.42	0.36		0.42	0.32		0.48	0.35		0.47	0.30
ETR	197.27	85.24		191.75	77.42		236.78	109.17		225.14	108.06		210.93	103.98		208.56	92.21

**Table 4.** Pairwise correlations among the traits under drought stress (above diagonal) and under control treatment (below diagonal)

	SPAD	LA	LN	PH	LRL	RN	LDW	SDW	SHDW	RDW	RSR	TDW	RL	RSA	RD	RV	NRT	NRF	NRC	Phot	F_vʹ_/F_mʹ_	ETR
SPAD	1.000	−0.003^NS^	0.120^NS^	−0.081^NS^	0.006^NS^	0.102^NS^	0.194^NS^	0.197^NS^	−0.205^NS^	−0.045^NS^	−0.043^NS^	0.007^NS^	−0.085^NS^	−0.132^NS^	−0.118^NS^	−0.163^NS^	0.216^NS^	−0.100^NS^	−0.116^NS^	0.030^NS^	0.240^NS^	0.393^NS^
LA	0.197^NS^	1.000	−0.231^NS^	0.231^NS^	−0.173^NS^	−0.084^NS^	0.667**	0.468^NS^	0.197^NS^	−0.164^NS^	−0.099^NS^	0.440*	0.239^NS^	0.020^NS^	−0.434*	−0.153^NS^	0.503*	0.129^NS^	0.106^NS^	−0.116^NS^	0.245^NS^	0.205^NS^
LN	0.059^NS^	0.618**	1.000	0.448*	−0.069^NS^	0.411*	−0.088^NS^	0.805^NS^	0.227^NS^	0.019^NS^	0.828*	0.058^NS^	−0.270^NS^	−0.175^NS^	0.183^NS^	−0.084^NS^	−0.366^NS^	−0.217^NS^	−0.124^NS^	−0.175^NS^	0.031^NS^	0.100^NS^
PH	0.086^NS^	0.580**	0.642**	1.000	−0.239^NS^	0.043^NS^	0.096^NS^	0.833*	0.678**	−0.375^NS^	0.681^NS^	0.161^NS^	−0.212^NS^	−0.339^NS^	−0.327^NS^	−0.399^NS^	−0.090^NS^	−0.250^NS^	−0.146^NS^	−0.422*	0.076^NS^	0.468^NS^
LRL	−0.268^NS^	0.035^NS^	0.192^NS^	−0.065^NS^	1.000	0.055^NS^	0.140^NS^	0.836*	−0.246^NS^	0.374^NS^	0.889*	0.196^NS^	0.087^NS^	0.172^NS^	0.201^NS^	0.211^NS^	0.072^NS^	0.053^NS^	−0.054^NS^	−0.029^NS^	−0.206^NS^	0.711^NS^
RN	−0.061^NS^	0.583**	0.194^NS^	−0.014^NS^	0.149^NS^	1.000	0.105^NS^	0.593^NS^	0.219^NS^	0.091^NS^	0.236^NS^	0.225^NS^	−0.251^NS^	−0.247^NS^	−0.030^NS^	−0.213^NS^	−0.381^NS^	−0.203^NS^	0.139^NS^	−0.422*	−0.424*	0.672^NS^
LDW	0.212^NS^	0.955**	0.614**	0.458*	0.020^NS^	0.559**	1.000	0.182^NS^	0.235^NS^	−0.051^NS^	−0.033^NS^	0.745**	0.033^NS^	−0.116^NS^	−0.321^NS^	−0.226^NS^	0.469*	−0.049^NS^	−0.119^NS^	−0.244^NS^	0.049^NS^	0.571^NS^
SDW	0.296^NS^	0.553^NS^	0.484^NS^	0.338^NS^	0.281^NS^	0.749^NS^	0.730^NS^	1.000	0.823*	0.853*	0.786^NS^	0.631^NS^	−0.509^NS^	−0.552^NS^	−0.390^NS^	−0.556^NS^	−0.393^NS^	−0.535^NS^	−0.349^NS^	0.364^NS^	−0.106^NS^	−0.340^NS^
SHDW	0.140^NS^	0.878**	0.617**	0.466*	0.080^NS^	0.557**	0.942**	0.877*	1.000	−0.197^NS^	0.545^NS^	0.508*	−0.268^NS^	−0.363^NS^	−0.262^NS^	−0.386^NS^	−0.083^NS^	−0.327^NS^	−0.171^NS^	−0.495*	−0.133^NS^	0.093^NS^
RDW	0.061^NS^	0.672**	0.533**	0.218^NS^	0.079^NS^	0.678**	0.755**	0.927**	0.826**	1.000	0.886*	0.458*	0.474*	0.689**	0.606**	0.771**	0.052^NS^	0.513*	0.429*	0.001^NS^	−0.003^NS^	−0.656^NS^
RSR	0.248^NS^	−0.301^NS^	−0.171^NS^	−0.127^NS^	−0.283^NS^	0.911*	−0.241^NS^	0.450^NS^	−0.026^NS^	0.198^NS^	1.000	0.624^NS^	−0.232^NS^	−0.123^NS^	0.076^NS^	−0.061^NS^	−0.358^NS^	−0.398^NS^	−0.335^NS^	0.478^NS^	−0.064^NS^	−0.403^NS^
TDW	0.172^NS^	0.922**	0.627**	0.444*	0.052^NS^	0.597**	0.982**	0.923**	0.983**	0.845**	0.075^NS^	1.000	0.173^NS^	0.156^NS^	0.018^NS^	0.120^NS^	0.303^NS^	0.114^NS^	0.091^NS^	−0.393^NS^	−0.031^NS^	−0.230^NS^
RL	0.385^NS^	0.539**	0.454*	0.249^NS^	0.015^NS^	0.414*	0.631**	0.622^NS^	0.696**	0.725**	0.358^NS^	0.693**	1.000	0.905**	−0.048^NS^	0.704**	0.670**	0.949**	0.782**	−0.060^NS^	0.353^NS^	−0.237^NS^
RSA	0.206^NS^	0.707**	0.537**	0.292^NS^	0.097^NS^	0.707**	0.753**	0.697^NS^	0.809**	0.923**	0.704^NS^	0.827**	0.841**	1.000	0.381^NS^	0.939**	0.442*	0.937**	0.670**	0.033^NS^	0.242^NS^	−0.055^NS^
RD	0.022^NS^	0.692**	0.466*	0.262^NS^	0.159^NS^	0.795**	0.674**	0.401^NS^	0.697**	0.841**	0.807^NS^	0.732**	0.500*	0.886**	1.000	0.674**	−0.397^NS^	0.135^NS^	−0.123^NS^	0.218^NS^	−0.151^NS^	0.463^NS^
RV	0.119^NS^	0.695**	0.530**	0.272^NS^	0.114^NS^	0.756**	0.726**	0.670^NS^	0.783**	0.930**	0.765^NS^	0.804**	0.722**	0.980**	0.951**	1.000	0.200^NS^	0.800**	0.492*	0.112^NS^	0.130^NS^	0.088^NS^
NRT	0.193^NS^	−0.269^NS^	0.003^NS^	−0.077^NS^	−0.028^NS^	−0.372^NS^	−0.175^NS^	−0.244^NS^	−0.087^NS^	−0.149^NS^	−0.270^NS^	−0.145^NS^	0.284^NS^	−0.126^NS^	−0.472*	−0.237^NS^	1.000	0.533**	0.250^NS^	0.093^NS^	0.591**	0.475^NS^
NRF	0.209^NS^	0.696**	0.550**	0.337^NS^	0.047^NS^	0.622**	0.753**	0.682^NS^	0.828**	0.863**	0.749^NS^	0.825**	0.889**	0.947**	0.755**	0.896**	0.037^NS^	1.000	0.801**	−0.081^NS^	0.261^NS^	−0.165^NS^
NRC	0.131^NS^	0.432*	0.339^NS^	0.194^NS^	0.001^NS^	0.365^NS^	0.504*	0.670^NS^	0.585**	0.588**	0.333^NS^	0.565**	0.825**	0.650**	0.347^NS^	0.536**	0.154^NS^	0.802**	1.000	−0.281^NS^	0.071^NS^	−0.586^NS^
Phot	−0.144^NS^	0.044^NS^	−0.130^NS^	0.086^NS^	0.011^NS^	0.267^NS^	−0.109^NS^	0.362^NS^	−0.040^NS^	0.057^NS^	0.829*	−0.064^NS^	−0.042^NS^	0.108^NS^	0.221^NS^	0.157^NS^	−0.046^NS^	0.093^NS^	0.029^NS^	1.000	0.471*	0.338^NS^
F_vʹ_/F_mʹ_	−0.275^NS^	−0.257^NS^	−0.156^NS^	−0.026^NS^	0.137^NS^	0.020^NS^	−0.421*	−0.694^NS^	−0.365^NS^	−0.282^NS^	−0.385^NS^	−0.397^NS^	−0.406*	−0.296^NS^	−0.125^NS^	−0.226^NS^	−0.082^NS^	−0.333^NS^	−0.303^NS^	0.752**	1.000	0.851*
ETR	0.328^NS^	0.191^NS^	0.233^NS^	0.013^NS^	0.044^NS^	0.971**	0.357^NS^	0.840*	0.540^NS^	0.596^NS^	0.790^N^	0.608^NS^	0.629^NS^	0.783^NS^	0.639^NS^	0.781^NS^	−0.072^NS^	0.813*	0.562^NS^	0.651^NS^	−0.714^NS^	1.000

* = *p* < 0.01 and ** = *p* < 0.001. Abbreviations referred from Table 3.

**Table 5.** Classification of corn genotypes into three drought response groups based on CDSRI along with individual scores in parenthesis

Sensitive reaction	Moderate reaction	Tolerant reaction
25.34–26.50	26.50–27.66	27.66–28.82
P1319 (25.21)	P1498 (26.89)	DKC 6581 (28.32)
N59B-3111A (25.34)	DKC6697 (26.85)	N61X-3110 (28.02)
